# *Escherichia coli* O157:H7 suppresses host autophagy and promotes epithelial adhesion via Tir-mediated and cAMP-independent activation of protein kinase A

**DOI:** 10.1038/cddiscovery.2017.55

**Published:** 2017-10-02

**Authors:** Yansong Xue, Min Du, Haiqing Sheng, Carolyn J Hovde, Mei-Jun Zhu

**Affiliations:** 1 School of Food Science, Washington State University, Pullman, WA 99164, USA; 2 Department of Animal Science, Washington State University, Pullman, WA 99164, USA; 3 School of Food Science, University of Idaho, Moscow, ID 83844, USA

## Abstract

Autophagy is a pivotal innate immune response that not only degrades cytosolic components, but also serves as one of the critical antimicrobial mechanisms eliminating intracellular pathogens. However, its role in host defense against extracellular pathogens is largely unknown. Here we showed that *E. coli* O157:H7 altered autophagy to evade host defense and facilitate adhesion. Enhancing host cell autophagy with tumor necrosis factor (TNF), host starvation or rapamycin reduced the adherence of *E. coli* O157:H7 to HT-29 cells. As a key regulator of autophagy, protein kinase A (PKA) was activated by *E. coli* O157:H7 infection. PKA inhibition by H89 abrogated *E. coli* O157:H7 inhibition of autophagy and prevented bacterial epithelial adhesion. Thus, PKA had a mediatory role in blocking autophagy and *E. coli* O157:H7 epithelial adhesion. Furthermore, deletion of translocated intimin receptor (*tir*) prevented PKA activation, whereas ectopic *tir* expression in a Δ*tir* mutant strain restored its ability to activate PKA and inhibited autophagy in host cells. This indicated that Tir and PKA played pivotal roles in manipulating host autophagy during infection. Consistent with autophagy inhibition, *E. coli* O157:H7 infection inhibited endoplasmic reticulum (ER) stress in HT-29 cells, which was reversed by TNF, starvation, or H89 treatment. Additionally, *E. coli* O157:H7-induced PKA activation suppressed extracellular signal-regulated kinase 1/2 (ERK1/2) activation and enhanced phosphatidylinositol 3-kinase/Akt (PI3K/Akt) signaling, thereby repressing autophagic signaling. Conversely, PKA inhibition prevented downregulation of ERK1/2 signaling due to *E. coli* O157:H7 infection. In summary, *E. coli* O157:H7 inhibited host autophagy via Tir-mediated PKA activation that favored bacterial persistence on intestinal epithelial cell surfaces.

## Introduction

Autophagy is a crucial process for degrading intracellular proteins and organelles^1^ and, recently, it is recognized as a critical self-defense mechanism to microbial infection.^2^ Canonical autophagy is characterized by the formation of a double-membrane autophagosome that involves over 35 autophagy-related proteins (Atgs), including a commonly used autophagosome marker, microtubule-associated protein 1 light chain-3B (LC3B).^3^ The autophagosome fuses with lysosomes, resulting in the degradation of engulfed components.^3^

Invasive pathogens have evolved different mechanisms to evade the trap by autophagy. *Listeria monocytogenes* and *Shigella flexneri* can escape autophagic capture by blocking the recruitment of autophagic proteins such as Beclin1 and Atg7 and subsequently inhibiting the maturation of the phagosomes.^4,5^ On the other hand, autophagic vacuole may be utilized by pathogens as a shelter that protects the invading microorganisms from lysis and facilitates their growth inside the host cells.^6^ *Porphyromonas gingivalis* can replicate in vacuoles by degrading autophagic proteins into amino acids that serve as nutritional sources.^7^ Importantly, some pathogens have evolved effector proteins to interfere with the autophagic clearance. *Salmonella* Type III secretion systems (T3SS) effector SseL (*Salmonella* secreted effector L) is able to deubiquitinate autophagic proteins for autophagosome formation and maturation, enabling resistance to lysosomal degradation.^8^ *Legionella* T3SS effector RavZ inhibits host autophagy through its cysteine protease activity.^9^ Thus, manipulation of the host autophagic process plays an important role in the pathogenesis of invasive pathogens. Up to now, however, the interaction of extracellular pathogens with host autophagy has been rarely explored.

*E. coli* O157:H7 is a major extracellular foodborne pathogen that produces Shiga toxin (Stx) and causes life-threatening hemolytic-uremic syndrome (HUS).^10^ In addition, it contains a chromosomal pathogenicity island, locus of enterocyte effacement (LEE), encoding T3SS apparatus and effectors that lead to intimate adhesion to host intestinal epithelial cells and formation of attaching and effacing (AE) lesions.^11^ Tir is one of the LEE-encoded effectors that is translocated into host cells via T3SS and embedded into the host cell membrane,^12^ acting as a receptor for the bacteria’s own outer membrane protein, intimin, further mediating intimate adherence to gut epithelial cells. In enteropathogenic *E. coli* (EPEC), the intracellular domain of Tir induces host signaling pathways including activation of cellular cyclic adenosine monophosphate (cAMP)/cAMP-dependent serine/threonine protein kinase A (PKA), that initiates cell membrane protein rearrangement.^13^ Interestingly, PKA has been implicated in suppressing autophagy. Loss of PKA enhances autophagic turnover of *Saccharomyces cerevisiae.*^14^ More recently, it is discovered that PKA directly phosphorylates Atg13 protein and disassociates it from autophagosome.^15^

Endoplasmic reticulum (ER) stress is a cellular response to sustained accumulation of unfolded or misfolded proteins in ER lumen. IRE1, PERK, and ATF6 are three major transducers of the ER stress that sense the stress and transduce signals to the nucleus or cytosol.^16^ Autophagy is induced as a novel signaling in response to ER stress.^17^ In IRE1-deficient cells, the autophagy induced by ER stress was inhibited, indicating that the IRE1 pathway is required for autophagy activation stimulated by ER stress.^17^ We hypothesize that *E. coli* O157:H7 adhesion activates PKA via Tir that suppresses autophagy in infected host cells, promoting pathogen survival and colonization on the host cell surface.

## Results

### *E. coli* O157:H7 infection subverted autophagy in HT-29 cells

**Figure 1:**
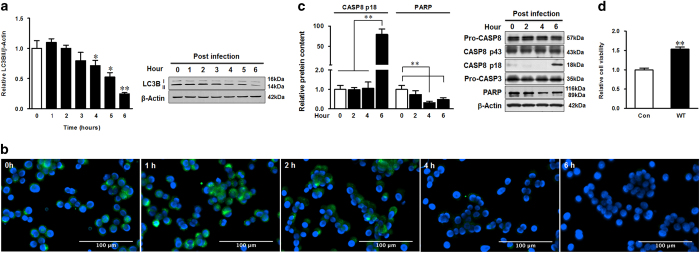
*E. coli* O157:H7 inhibits autophagy without causing apoptosis. (**a**) Immunoblotting analysis and densitometric quantification of band intensity of LC3B in HT-29 cells 0–6 h post infection of *E. coli* O157:H7 EDL933 WT strain. (**b**) Immunofluorescent staining for LC3B in HT-29 cells 0–6 h post infection of EDL933 WT strain. Blue: DAPI; green: LC3B. (**c**) Immunoblotting analysis and densitometric quantification of band intensity of caspase-8, caspase-3, and PARP in HT-29 cells 6 h post infection of EDL933 WT strain. (**d**) MTT test for HT-29 cells 4 h post infection of EDL933 WT strain. Means ±S.E.M.; *n*=4. *P<0.05; **P<0.01.

During the process of autophagy, LC3B-I is converted to LC3B-II by phosphatidylethanolamine conjugation. Thus, tracking the change of LC3B-II is indicative of autophagic activity.^18,19^ HT-29 cells were incubated with *E. coli* O157:H7 for 0–6 h before immunoblotting with LC3B antibody. The LC3B-II level gradually decreased at 4 h post infection (Figure 1a) that was further demonstrated by immunofluorescent staining (Figure 1b), showing that the LC3B-positive staining started to decrease at 4 h of infection (Figure 1b). To exclude the possibility of cell apoptosis contributing to the reduced LC3B-II level, we analyzed the caspases by immunoblotting. Although caspase-8 was cleaved into p18 after 6 h, caspase-3 was not activated (no activated fragments were detected) during *E. coli* O157:H7 infection (Figure 1c). *E. coli* O157:H7 infection even blocked the activation of PARP, a downstream substrate of caspase-3 (Figure 1c). The MTT assay, which measures cell viability by mitochondrial metabolic capability, further showed that *E. coli* O157:H7 infection enhanced cell metabolism in HT-29 cells (Figure 1d).

**Figure 2:**
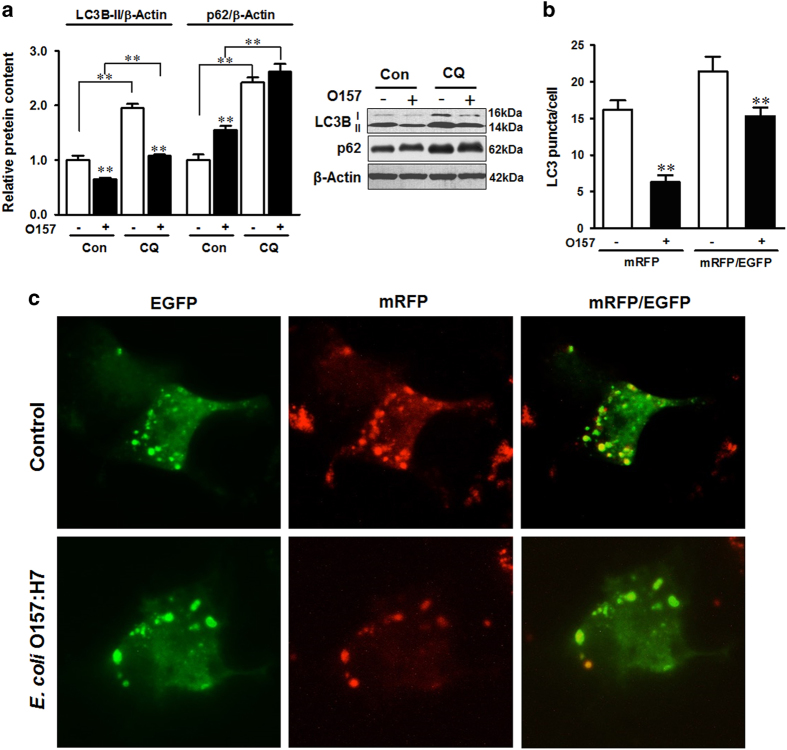
*E. coli* O157:H7 inhibits autophagic flux. (**a**) Autophagic degradation activity is inhibited by EDL933 WT strain as indicated by altered LC3B and p62 protein levels 4 h post infection. CQ: cells were treated with 20 *μ*M CQ during the course of infection. (**b** and **c**) Microscopic analysis of HT-29 cells transfected with tfLC3 (tandem fluorescent tagged LC3B) plasmids 4 h post infection. mRFP: Red puncta represent autophagolysosome that is late stage of autophagy tagged with mRFP; mRFP/EGFP: Yellow puncta represent autophagosome that is double tagged with both mRFP and EGFP; Control: HT-29 cells with tfLC3 without infection; *E. coli* O157:H7: HT-29 cells with tfLC3 challenged with EDL933 WT strain. Images were taken at ×400 magnification. Means±S.E.M.; *n*=4. **P<0.01.

To further demonstrate the decrease of LC3B-II was not due to the LC3B degradation by activated autophagosome, we analyzed autophagy flux in the presence of autolysosome acidification inhibitor, chloroquine (CQ), which inhibits the degradation process of autophagy, to see whether LC3B-II was indeed decreased by infection (Figure 2a). CQ selectively impaired lysosomal acidification and blocked the basal degradation of LC3B-II. Thus, the LC3B-II levels in cells without *E. coli* O157:H7 infection were increased by CQ treatment (Figure 2a). However, *E. coli* O157:H7 decreased LC3B-II level in the presence of CQ (Figure 2a), indicating that autophagic flux was blocked upon infection. In addition, infection and CQ treatment caused increased accumulation of p62 (Figure 2a). We further transfected the cells with tfLC3 plasmid tandem fluorescent-tagged with mRFP (red) and EGFP (green).^20^ In the initial stage, autophagosomes were double-tagged (mRFP and EGFP) with a yellow fluorescence.^3^ In late stage, the fusion of autophagosomes with lysosomes results in an acidic atmosphere and leads to the degradation of EGFP fluorescence and consequently emits red fluorescence.^21^ We found that infection decreased both the number of yellow and red puncta (Figures 2b and c), indicating *E. coli* O157:H7 interfered with autophagy at the initial stage of autophagy and decreased the formation of autophagosome. Together, this suggested that *E. coli* O157:H7 infection subverted autophagy in HT-29 cells.

### TNF, starvation, or rapamycin inhibited *E. coli* O157:H7 adhesion by inducing autophagy

**Figure 3:**
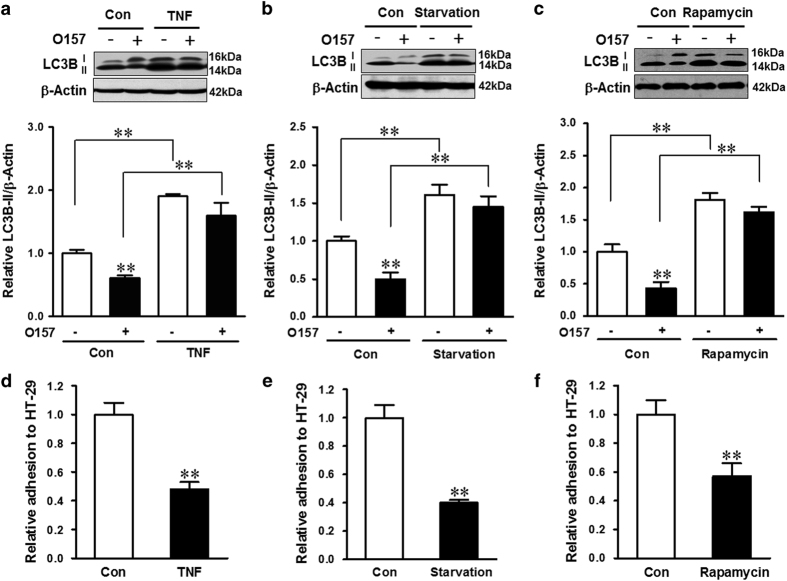
*E. coli* O157:H7 adhesion is inhibited by TNF, starvation, or rapamycin treatment. (**a**–**c**) Immunoblotting analysis and densitometric quantification of band intensity of LC3B in HT-29 cells pretreated with TNF (**a**), starvation (**b**), or rapamycin (**c**) followed with coincubation with EDL933 WT strain for 4 h. (**d**–**f**) Bacterial adhesion assay of EDL933 WT strain. HT-29 cells that were pretreated with TNF (**d**), starvation (**e**), or rapamycin (**f**) and followed with coincubation with EDL933 WT strain for 4 h. Means±S.E.M.; *n*=4. **P<0.01.

Tumor necrosis factor (TNF) is reported to activate autophagy by elevation of LC3B-II and augmentation of autophagosome formation in human primary myoblasts and intestinal epithelial cells.^22,23^ Similarly, TNF treatment resulted in enhanced LC3B-II in HT-29 cells (Figure 3a). The activation of autophagy was correlated with reduced *E. coli* O157:H7 adhesion to host cells (Figure 3d). To further examine the role of autophagy in mediating *E. coli* O157:H7 adherence, HT-29 cells were subjected to starvation or treated with rapamycin, two well-known autophagy activators. Both treatments increased the LC3B-II level (Figures 3b and c), indicating the elevated autophagy. Correspondingly, *E. coli* O157:H7 adhesions to host cells were both reduced (Figures 3e and f). In addition, CQ treatment significantly increased the bacterial adhesion (Supplementary Figure S1). These data, in combination, showed that autophagy inhibited bacterial adhesion to host cells.

### Tir mediated *E. coli* O157:H7-induced host autophagy inhibition

**Figure 4:**
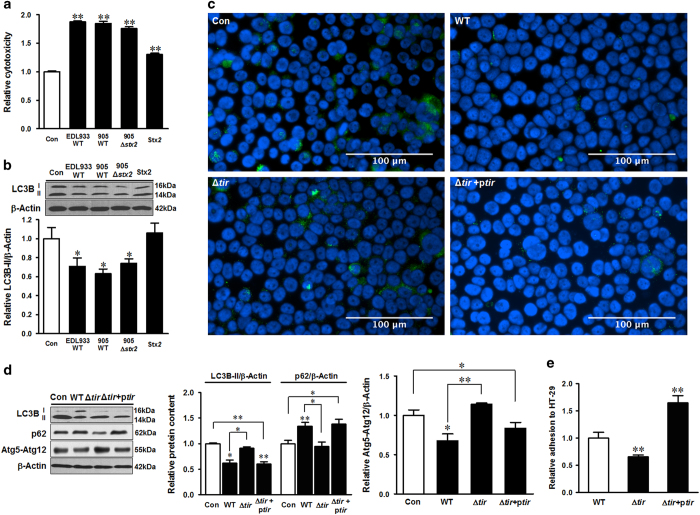
Tir participates in the inhibition of autophagy. Vero cell cytotoxicity induced by *E. coli* O157:H7 strains or purified Stx2 (**a**) and immunoblotting analysis of LC3B 4 h post coincubation with *E. coli* O157:H7 strains or Stx2 (**b**). Con: HT-29 cells without challenges; EDL993 WT: *E. coli* O157:H7 EDL933 wild-type strain; 905WT: *E. coli* O157:H7 905 wild-type strain; 905Δ*stx2*: *E. coli* O157:H7 905 stx2 deletion mutant strain; Stx2: purified Stx2. (**c** and **d**) Immunofluorescent staining and immunoblotting analysis of LC3B, p62, and Atg5-Atg12 conjugate in control HT-29 cells without infection (Con) or HT-29 cells 4 h post infection of EDL933 WT strain, *tir* knockout strain (Δ*tir*), or *tir* complementation strain (Δ*tir*+p*tir*). Blue: DAPI; green: LC3B. (**e**) The adhesion assay of *E. coli* O157:H7 EDL933 WT strain, tir knockout strain (Δ*tir*), or *tir* complementation strain (Δ*tir*+p*tir*) to HT-29 cells. Means±S.E.M.; *n*=4. *P<0.05; **P<0.01.

Autophagy is commonly activated by intracellular bacteria; however, *E. coli* O157:H7 is known as an extracellular pathogen. To confirm that autophagy was not affected by internalized *E. coli* O157:H7, we conducted an internalization assay and found the proportion of internalized bacteria were 6.15E−05% of the initial inoculation (Supplementary Table S1). This low level was unlikely to subvert host autophagy. To explore the major factors contributing to host autophagy inhibition, we analyzed the potential role of Stx2, the major virulence factor of *E. coli* O157:H7, in *E. coli* O157:H7 mediated autophagy inhibition. Vero cells or HT-29 cells were infected with *E. coli* O157:H7 EDL933 WT strain, *E. coli* O157:H7 905 WT strain and its isogenic Stx2 deletion mutant 905 strain (905 *Δstx2*), or treated with purified Stx2. The vero cell cytotoxicity assay confirmed that these strains were cytotoxic to the host cells (Figure 4a). However, HT-29 cells infected with EDL933 WT strain, containing both Stx1 and Stx2, *E. coli* O157:H7 905 WT strain, containing only Stx2, or 905 *Δstx2* mutant strain showed the similar extent of inhibition of LC3B-II level in HT-29 cells (Figure 4b). Furthermore, purified Stx2 did not alter autophagy activity in HT-29 cells (Figure 4b). These data showed that Stx2 had no major role in *E. coli* O157:H7-mediated autophagy inhibition.

Tir, a transmembrane virulence factor of *E. coli* O157:H7, plays a crucial role in bacterial adhesion to epithelial cells and induces pedestal formation on host cell membrane.^24^ Although Tir manages host actin rearrangement, we did not find significant changes of *β*-actin protein level during infection. To demonstrate its role in autophagy, HT-29 cells were infected with *E. coli* O157:H7 EDL933 WT or *tir* deletion mutant (*Δtir*) strains, respectively. EDL933 WT decreased both LC3B-II and Atg5/Atg12 levels, whereas increased the level of p62 (Figure 4d). Deletion of *tir* abolished *E. coli* O157:H7 ability to inhibit autophagy (Figure 4d); complementing *tir* in the Δ*tir* strain restored autophagy inhibition (Figures 4c and d), as well as the epithelial adhesion (Figure 4e). Data showed that Tir mediated the blockage of autophagy during *E. coli* O157:H7 infection.

### Tir-mediated autophagy inhibition was dependent on PKA

**Figure 5:**
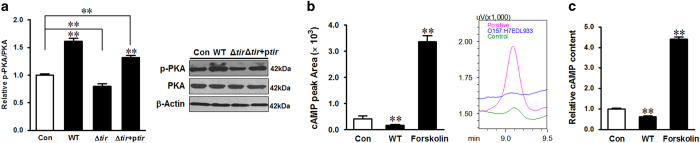
Tir mediates *E. coli* O157:H7-induced PKA activation in a cAMP-independent way. (**a**) Immunoblotting analysis and densitometric quantification of band intensity of PKA in HT-29 cells 4 h post infection of *E. coli* O157:H7 strains; Con: HT-29 cells without infection; WT: HT-29 cells infected with *E. coli* O157:H7 EDL933 WT strain; Δ*tir*: HT-29 cells infected with EDL933 *tir* mutant strain; Δ*tir*+p*tir*: HT-29 cells infected with EDL933 *tir* complementation strain. (**b** and **c**) cAMP level in HT-29 cells without infection or 4 h post infection of EDL933 WT strain measured by HPLC and ELISA. Forskolin was used as a positive control for cAMP activation. Means±S.E.M.; *n*=4. **P<0.01.

EPEC infection activates PKA that phosphorylates Tir at different sites in the N terminus.^25^ In HT-29 cells, *E. coli* O157:H7 infection induced robust activation of PKA, whereas Δ*tir* mutant failed to stimulate PKA (Figure 5a). Complementation of Δ*tir* with ectopic expression of *tir* recovered the ability to activate PKA (Figure 5a). To determine whether PKA activation is cAMP dependent, we measured the cAMP level post infection. Interestingly, in spite of PKA activation, *E. coli* O157:H7 infection decreased the cAMP level as measured by both HPLC (Figure 5b) and ELISA (Figure 5c). These data suggested that Tir-induced PKA activation was independent of cAMP.

**Figure 6:**
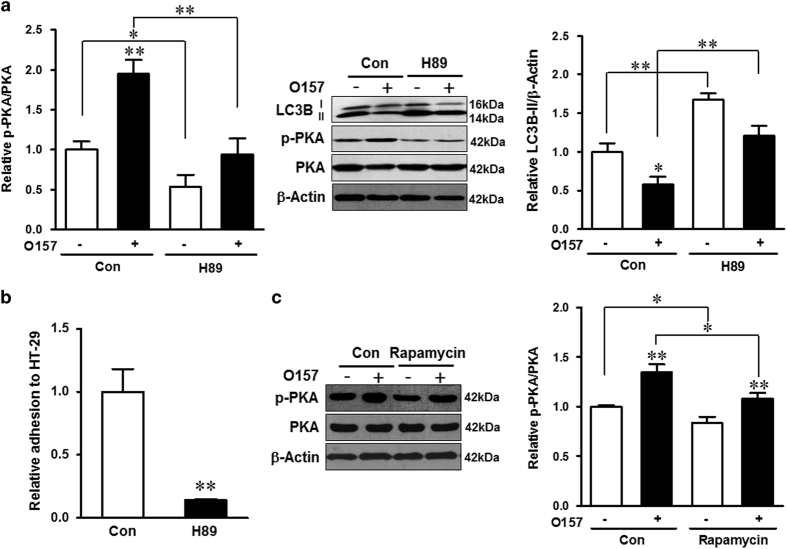
*E. coli* O157:H7 induces autophagy inhibition in a PKA-dependent way. (**a**) Immunoblotting analysis and densitometric quantification of band intensity of phosphorylated PKA (p-PKA), total PKA, and LC3B in HT-29 cells coincubated with H89 and challenged with EDL933 WT strain. (**b**) Bacterial adhesion assay of EDL933 WT strain to HT-29 cells coincubated with H89 (H89) or without H89 (Con). (**c**) Immunoblotting analysis and densitometric quantification of band intensity of phosphorylated and total PKA in HT-29 cells coincubated with or without rapamycin and challenged with or without EDL933 WT strain. Means±S.E.M.; *n*=4. *P<0.05; **P<0.01.

To examine whether PKA activation was sufficient to inhibit autophagy, H89, a PKA inhibitor, was used to inhibit PKA activity. H89 potently inhibited *E. coli* O157:H7-mediated PKA activation, which was coupled with robust elevation of the LC3B-II level (Figure 6a), and attenuated bacterial adhesion to epithelial cells (Figure 6b). Interestingly, rapamycin treatment blocked the activation of PKA in both infected and noninfected cells (Figure 6c). Thus, PKA at least partially mediated *E. coli* O157:H7-induced autophagy inhibition as well as epithelial adhesion to HT-29 cells.

### ERK signaling was associated with *E. coli* O157:H7-induced autophagy inhibition

**Figure 7:**
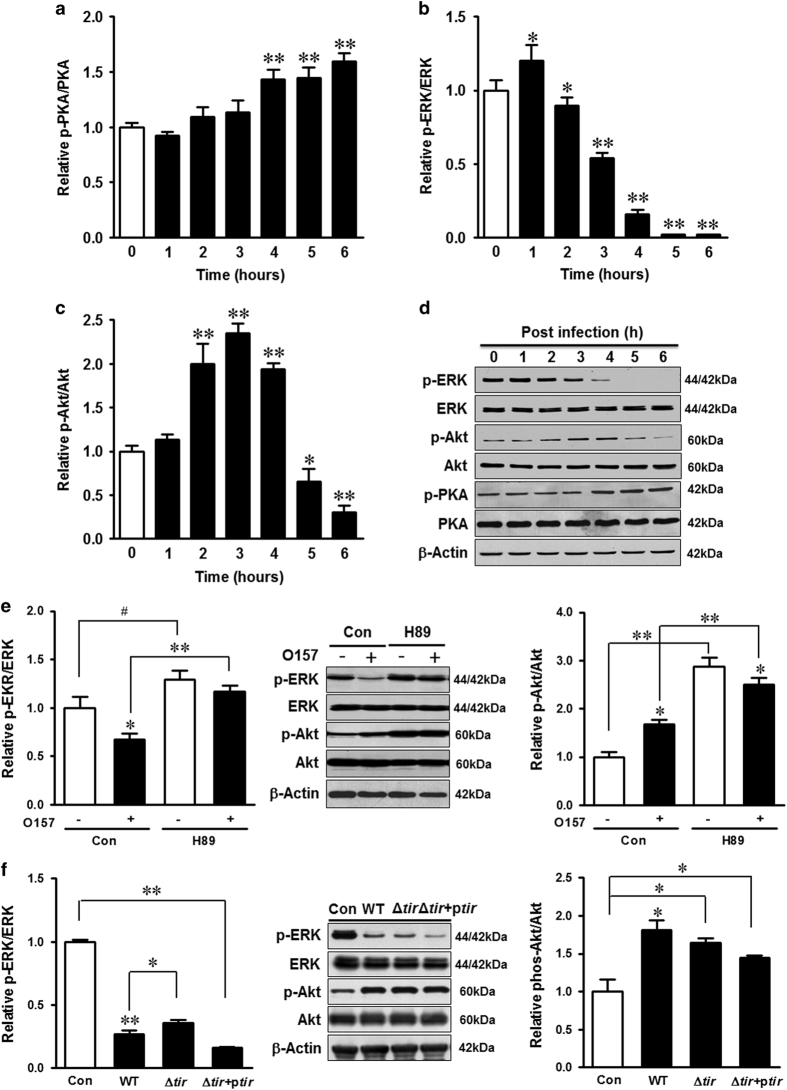
Infection causes the downregulation of ERK1/2 signaling. (**a**–**d**) Immunoblotting analysis and densitometric quantification of band intensity of phosphorylated and total PKA, ERK1/2, and Akt in HT-29 cells 0–6 h post infection of EDL933 WT strain. (**e**) Immunoblotting analysis and densitometric quantification of band intensity of phosphorylated and total ERK1/2 and Akt in HT-29 cells coincubated with or without H89 and challenged with or without EDL933 WT strain for 4 h. (**f**) Immunoblotting analysis and densitometric quantification of band intensity of phosphorylated and total ERK1/2 and Akt in HT-29 cells at 4 h post infection of EDL933 strains; Con: HT-29 cells without infection; WT: HT-29 cells infected with EDL933 WT strain; Δ*tir*: HT-29 cells infected with EDL933 *tir* mutant strain; Δ*tir*+p*tir*: HT-29 cells infected with EDL933 *tir* complementation strain. Means±S.E.M.; *n*=4. ^#^*P*<0.10; *P<0.05; **P<0.01.

To further explore the underling pathway, phosphatidylinositol-3-kinase (PI3K)/Akt and extracellular signal-regulated kinase-1/2 (ERK1/2) activities were analyzed upon *E. coli* O157:H7 infection. Accompanying with the inhibition of autophagy and activation of PKA in response to *E. coli* O157:H7 infection (Figure 7a), ERK1/2 phosphorylation was inhibited (Figure 7b), whereas Akt phosphorylation was increased during the first 4 h post infection (Figure 7c). Because PKA activation can negatively regulate ERK,^26^ we examined whether ERK inhibition during *E. coli* O157:H7 infection was PKA dependent. Interestingly, in the presence of PKA inhibitor, *E. coli* O157:H7 could no longer suppress ERK. These data suggested that PKA inhibited ERK and repressed autophagic signaling (Figure 7e). PKA inhibition enhanced Akt activation in both non-infected and infected cells; infection inhibited Akt activation in the presence of H89 (Figure 7e). In addition, as compared with that of HT-29 cells challenged with EDL933 WT strain, the phosphorylation of ERK was elevated or suppressed in HT-29 cells infected with Δ*tir* strain or *tir* complement Δ*tir* strain, respectively (Figure 7f).

### ER stress was inhibited upon *E. coli* O157:H7 infection

**Figure 8:**
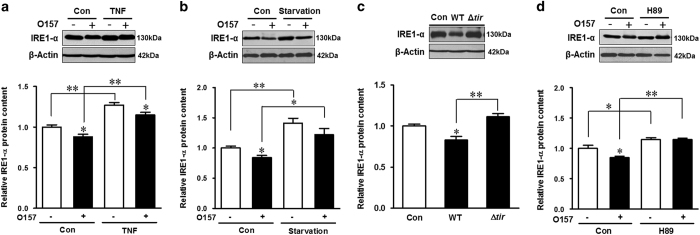
*E. coli* O157:H7 infection inhibits ER stress in HT-29 cells. (**a** and **b**) Immunoblotting analysis and densitometric quantification of band intensity of IRE1-α in HT-29 cells pretreated with TNF (**a**) or starvation (**b**) and coincubated with EDL933 WT strain for 4 h. (**c** and **d**) Immunoblotting analysis and densitometric quantification of band intensity of IRE1-*α* in HT-29 cells infected with EDL933 WT strain and its *tir* mutant strain (**c**) or incubated with or without H89 and challenged with or without EDL933 WT strain (**d**). Means±S.E.M.; *n*=4. *P<0.05; **P<0.01.

In host, the autophagy system is activated in response to ER stress.^17^ IRE1-*α* has an important role in inducing autophagy under ER stress.^17^ This prompted us to postulate that *E. coli* O157:H7 may inhibit autophagy through blocking the ER stress response. Indeed, we found that *E. coli* O157:H7 induced autophagy inhibition accompanied with decreased IRE1-*α* content (Figures 8a and b). In addition, associated with enhanced autophagy, either TNF or starvation treatments induced ER stresses, as indicated by enhanced IRE1-*α* level, which could mask the suppressive effect of *E. coli* O157:H7 on IRE1-*α* protein content (Figures 8a and b). However, *E. coli* O157:H7 was unable to inhibit ER stress in cells with PKA abrogated by PKA inhibitor (Figure 8d). In addition, the *E. coli* O157:H7 *tir* deletion mutant was not able to inhibit ER stress (Figure 8c). Data collectively indicated that Tir could also inhibit ER stress in a PKA-dependent manner.

## Discussion

Although autophagy is an important defense mechanism to intracellular pathogens, many pathogenic bacteria have evolved strategies to evade this immune response. However, whether extracellular pathogens also manipulate autophagy for adhesion and survival on host cells is unclear.

### *E. coli* O157:H7 infection subverted autophagy in HT-29 cells via Tir

It has been reported that RavZ of *Legionella pneumophila* irreversibly inactivates LC3B.^9^ We think that at the first 2 h, the cells were activated by infection and LC3B-I was converted to LC3B-II. With the infection time increased, bacteria started to interfere with the LC3B-II level and led to the decrease of autophagosomes. Autophagosome is the initial stage of autophagy. In late stage, autophagosome fuses with lysosome to form autophagolysosome. CQ inhibits the fusion of autophagosomes with lysosomes which causes the accumulation of autophagosomes. In the current study, infection caused the decrease of LC3B-II level even with the treatment of CQ, indicating that *E. coli* O157:H7 blocked or even damaged the formation of autophagosomes. In AIEC (adherent invasive *E. coli*)-infected neutrophils, the LC3B-II level has no further increase when treated with lysosomal protease inhibitors E64d and Pepstatin, indicating the impaired autophagic flux.^27^ The autophagic flux was further confirmed by transfection of tfLC3. In late stage, the acidic autophagolysosomes degraded EGFP (green fluorescence) turning into red fluorescence. Infection decreased the yellow puncta (autophagosomes), indicating that *E. coli* O157:H7 interfered with initial stage of autophagy. The red fluorescence, which showed autophagolysosomes, was subsequently reduced because of the decrease of autophagosomes. Our data showed that infection reduced autophagosome formation by decreasing LC3B level that occurred before the fusion with lysosomes. Stx is reported to activate autophagy in a host cell-dependent manner.^28^ The purified Stx1 and Stx2 trigger autophagy in human monocyte-derived macrophages and THP-1 myeloid cells but not in human peripheral blood monocytes.^28^ In order to determine the potential role of Stx1 and Stx2 in autophagy, we used 905 WT and its isogenic stx2 deletion mutant strain (905 Δ*stx2*) in addition to EDL933 WT strain. We detected a similar extent of inhibition of LC3B-II level in 905 WT strain containing only Stx2 as that of 905 Δ*stx2* mutant strain and the EDL933 strain containing both Stx1 and Stx2. This indicates that Stx1 or Stx2 is not a major factor for the inhibited autophagy activity. In addition, the purified Stx2 was not able to alter autophagy response in HT-29 cells, further confirming that Stx2 was not a major player in the observed autophagy inhibition. The overall effect on host autophagy may be due to other virulence factors. Tir is an important transmembrane virulence factor injected into host cells by *E. coli* O157:H7 T3SS mediating intimate association with host cells.^29^ Using EDL933 WT, Δ*tir*, and *tir* complementation strains, we found that Tir was one of the major players in autophagy inhibition upon *E. coli* O157:H7 infection.

### Tir was associated with autophagy inhibition in a PKA-dependent manner

It has been reported that Tir_EPEC_ has a mutual interaction with host PKA.^29^ PKA is activated upon EPEC infection, and this activation is attenuated in the *tir* mutant infected cells.^29^ Here we demonstrated that *E. coli* O157:H7 infection activated PKA signaling in epithelial cells, whereas *tir* deletion could not activate PKA, showing that Tir mediated PKA activation. We further found that PKA inhibitor H89 treatment led to a robust elevation of autophagy activation in HT-29 cells associated with attenuated EDL933 WT adhesion. These data collectively indicated that *E. coli* O157:H7, as an extracellular pathogen, inhibited host autophagy possibly through Tir and associated with PKA activation.

Much evidence indicates that the PKA pathway is utilized by intracellular pathogens to promote their survival.^30,31^ PKA activation due to increased cAMP levels inhibits phagosome–lysosome fusion and acidification that facilitates *Mycobacterium tuberculosis* survival and growth in macrophages.^32^ During *Salmonella* Typhimurium infection, treatment of epithelial cells with cAMP-elevating toxin from *Bacillus anthracis* or *Vibrio cholerae* results in decreased LC3B positive vacuoles and increased *S.* Typhimurium survival.^33^ Interestingly, we found that *E. coli* O157:H7-mediated PKA activation in HT-29 cells was independent of cAMP. In support of our finding, a previous study reported a cAMP-independent activation of PKA in human umbilical vein endothelial cells treated with Stx2B.^34^

### ERK was downstream target of activated PKA in *E. coli* O157:H7-induced autophagy inhibition

The ERK1/2 pathway positively regulates autophagy.^35^ Activation of ERK1/2 has been reported to be responsible for the autophagosome membrane recruitment and formation during autophagy in HT-29 cells.^36^ Pharmacological inhibition of ERK1/2 phosphorylation with U0126 or PD98059 results in a decrease in TNF-induced autophagy in MCF-7 breast cancer cells.^37^ Furthermore, ERK1/2 phosphorylation is blocked by either EPEC^38^ or EHEC^37,39^ infection. In our study, although the mRNA expressions of ERK1/2 were not altered by infection (Supplementary Figure S2), the protein level of phosphorylated ERK1/2 was decreased. We found that EDL993 Δ*tir* mutant strain was not able to suppress ERK1/2 signaling in HT-29 cells upon infection; neither was EDL993 WT able to suppress ERK1/2 signaling in HT-29 cells treated with PKA inhibitor, showing that the abrogated ERK1/2 activation was associated with *E. coli* O157:H7 Tir-induced PKA activation and autophagy inhibition. In support of our finding, Tir_EPEC_ is reported to interact with the cellular tyrosine phosphatase Src homology 2 (SHP-2)^40^ that facilitates the recruitment of SHP-2 to TRAF6 and downstream inactivation of ERK1/2.^40^ Collectively, *E. coli* O157:H7 Tir interacted with PKA and subsequently deactivated ERK1/2 activity that manipulated autophagy activation. Previously, we reported that TNF decreased *E. coli* O157:H7 adhesion partially through ERK activation.^39^ This study as well as a previous study,^23^ showed that TNF induced autophagy in HT-29 cells that inhibited pathogen adhesion. Consistently, we further found that starvation or rapamycin-induced autophagy reduced bacterial attachment. Collectively, these data showed that ERK1/2 inactivation was associated with autophagy inhibition upon *E. coli* O157:H7 infection.

### *E. coli* O157:H7 inhibited autophagy in HT-29 cells associated with ER stress

LC3B is extensively aggregated near the site of ER when cells are suffered with ER stress.^17^ ER stress is highly correlated with unfolded or misfolded proteins in ER, a potent stimulus of autophagy.^41^ Stx2 of *E. coli* O157:H7 is reported to stimulate ER stress by eukaryotic translation initiation factor 2 subunit 1 (EIF2S1) activation and stress protein recruitment,^42^ suggesting its possible involvement in autophagy activation in intestinal epithelial cells.^42^ However, our study found that *E. coli* O157:H7 infection upregulated the mRNA expression of IRE1-*α* (Supplementary Figure S2), whereas it decreased the protein content, indicating the block of the protein translation by infection. However, *tir* mutation abolished their ability to suppress IRE1-*α* protein, suggesting that Tir negatively regulated IRE1-*α* accumulation. Furthermore, the presence of PKA inhibitor abrogated IRE1-*α* inhibition by infection, indicating *E. coli* O157:H7 negatively regulated ER stress through PKA activation, and subsequently inhibited autophagy. We demonstrated that both TNF and serum starvation induced ER stress that was correlated with their upregulation of autophagy. In response to bacterial infection, activated ER stress helps host cells to eliminate the pathogen.^43^ Infection with herpes simplex virus (HSV) results in ER stress response that interferes with viral mRNA translation and protein synthesis.^44^ In addition, ER stress-induced apoptosis inhibits the growth of pathogens by ceasing their replication and promoting their phagocytosis by immune cells.^45^ Pathogens have evolved to manipulate this effect to halt host apoptosis in order to extend colonization.^46–48^ T3SS effector NleB1 of EPEC binds to host FAS-associated death domain (FADD), TNFR1-associated death domain protein (TRADD), and RIPK1 that prevents TNF-induced activation of caspase-8 with its *N*-acetylglucosamine transferase (GlcNAc) activity.^46–48^ This is confirmed in our study that although *E. coli* O157:H7 infection activated caspase-8 at 6 h post infection, they did not cause the cleavage of caspase-3 and even inhibited the cleavage of PARP. This could be a strategy of *E. coli* O157:H7 to block autophagy activation. Taken together, *E. coli* O157:H7 halted cell apoptosis and inhibited autophagy, both of which facilitated pathogen colonization and growth.

### Clinical implication

*E. coli* O157:H7 is a major foodborne pathogen causing life-threatening HUS. Epithelial colonization of *E. coli* O157:H7 is one of the key factors leading to serious illness. Antibiotics, which are commonly used to treat bacterial infection, are not applicable for *E. coli* O157:H7 infection because of their ability to stimulate Stx secretion that intensifies intoxication. Autophagy plays an important role in host defense against bacterial infection; in this study, we demonstrate that *E. coli* O157:H7 has evolved strategies to block host autophagic response partially via activation of PKA. Thus, the drugs/chemicals suppressing PKA activation during *E. coli* O157:H7 infection may provide potential therapies in reducing *E. coli* O157:H7 gut colonization and infection.

## Conclusions

**Figure 9:**
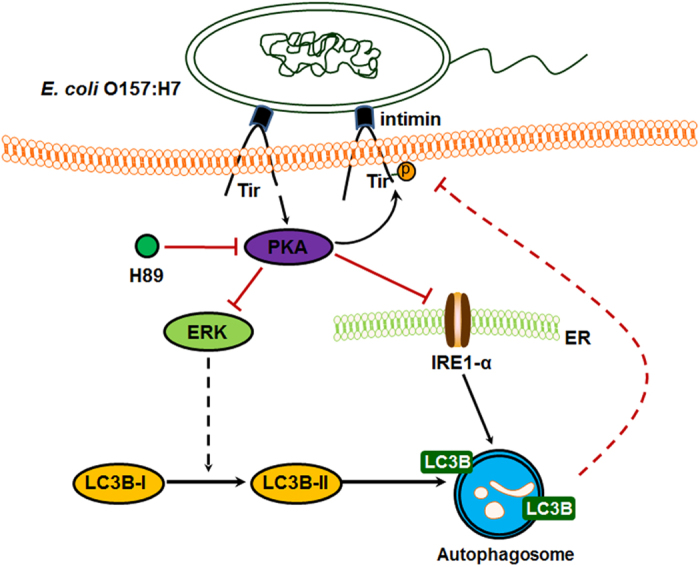
Model for host cell autophagy signaling induced by *E. coli* O157:H7. Infection of *E. coli* O157:H7 induces PKA activation that inhibits host autophagy activation, possibly through MAPK/ERK1/2 and ER stress signaling pathways. H89 is a specific inhibitor of PKA. The arrows indicate demonstrated effects. The blocking steps are indicated.

In summary, we illustrate that *E. coli* O157:H7 actively suppresses autophagy through a Tir-dependent mechanism that involves cAMP-independent PKA activation. The PKA pathway negatively regulates ERK signaling and ER stress and further attenuates the autophagic response. Activation of autophagy impairs the adhesion of *E. coli* O157:H7 to host cells (Figure 9).

## Materials and methods

### Cell line, media, and bacterial strains

The human colonic epithelial cell line HT-29 was obtained from the American Type Culture Collection (Manassas, VA, USA). HT-29 cells were cultured in Dulbecco’s modified Eagle’s medium (DMEM) (Sigma, St. Louis, MO, USA) supplemented with 10% fetal bovine serum (Sigma), 100 units/ml penicillin G, and 100 *μ*g/ml of streptomycin (Sigma) at 37 °C with 5% CO_2_. The *E. coli* O157:H7 EDL933 strain (WT) was obtained from the STEC center at Michigan State University and routinely grown in LB broth at 37 °C overnight with aeration. Shiga toxin type 2 (Stx2) was purchased from Toxin Technology (Sarasota, FL, USA). The *E. coli* O157:H7 905 wild-type strain (a human clinical isolate, only producing Stx2) and its isogenic Stx2 deletion mutant 905 strain and *E. coli* O157:H7 EDL933 *tir* deletion mutant strain (Δ*tir*) were from Dr. Hovde’s Lab at the University of Idaho (Moscow, ID, USA).^49^ pEHEC *tir* plasmid was a generous gift from Dr. Leong at Tufts University (Medford, MA, USA).^50^ EDL933Δ*tir* pEHEC *tir* strain was derived from *E. coli* O157:H7 EDL933Δ*tir* strain transformed with pEHEC *tir* plasmid.

### *E. coli* O157:H7 infection of colonic epithelial cells

HT-29 cells (1×10^6^ cells per well) were seeded in 12-well plates and cultured until they reached 80–90% confluence. For TNF and rapamycin treatments, cells were pretreated with 10 ng/ml TNF or 2 *μ*M rapamycin for 12 h,^51^ washed 3 times with PBS (pH 7.4), and challenged with 10^7^ CFU/ml *E. coli* O157:H7 (MOI 10 : 1). For the starvation assay, at 3 h post infection, cells were cultured in EBSS medium (Sigma) and infected for another 2 h. All treatments were maintained for the course of infection. HT-29 cells were collected for protein extraction at 0–6 h post *E. coli* O157:H7 infection at 37 °C with 5% CO_2_.

### Bacterial adhesion assay

Bacterial adhesion assay was conducted as described in our previous publication.^39^ Briefly, HT-29 cells (5×10^5^ cells per well) were seeded in a 24-well plate and cultured until 80–90% confluence. Cells were infected with *E. coli* O157:H7 EDL933 WT, Δ*tir*, or Δ*tir* pEHEC *tir* strains for 4 h. After infection, the cell monolayers were washed 3 times with PBS and lysed with 0.1% Triton X-100. Serial dilutions were plated on LB agar and bacterial colonies were counted after 18 h of incubation at 37 °C. The percentage of adhesion was calculated by dividing the number of colonies (recovered *E. coli* O157:H7) by the initial number of *E. coli* O157:H7 added and multiplied by 100.

### MTT test

The growth inhibition of *E. coli* O157:H7 against HT-29 cells was assessed by MTT (3-(4,5-dimethylthiazol-2-yl)-2,5-diphenyltetrazolium bromide) assay.^52^ HT-29 cells were cultured in 96-well plates for 24 h and infected with *E. coli* O157:H7 EDL933 WT strain as described above. Cell monolayers were washed with ice-cold PBS. Then, 100 *μ*l of MTT solution was added to each well and incubated for additional 4 h to form the formazan. At the end of the incubation period the medium was discarded and the formazan was dissolved with 100 *μ*l DMSO. Absorbance of converted dye was measured at a wavelength of 540 nm using a microplate reader (BioTek Synergy H1, VT).^52^

### Immunofluorescence staining

Infected HT-29 cell monolayers were fixed with 4% fresh prepared paraformaldehyde. Cells were washed with PBS 3 times and permeabilized in 0.1% Triton X-100 for 10 min. After blocking with 1.5% goat serum for 30 min, cells were incubated with anti-LC3B antibody (1 : 200, Cell Signaling, Beverly, MA, USA) overnight, followed by incubation for 1 h at room temperature with anti-rabbit Alexa Fluor 488 conjugate secondary antibody (Cell Signaling), and mounted with VECTASHIELD mounting medium with DAPI (Vector Lab, Burlingame, CA, USA). Positive puncta were visualized using EVOS FL fluorescence microscope (Life Technologies, Grand Island, NY, USA).

### ptfLC3 transfection and fluorescence microscopy

HT-29 cells seeded in a 24-well plate reached 70% confluent at the time of transfection. tfLC3 (Addgene plasmid 21074, Cambridge, MA, USA) plasmids are tandem fluorescent (mRFP and EGFP) tagged LC3B that are able to detect different stages of autophagy. The EGFP tag is acid sensitive whereas the mRFP tag is acid resistant. The double-tagged LC3B can be used to label both autophagosomes and autolysosomes. In the beginning, autophagosomes are tagged by both mRFP and EGFP, resulting in a yellow fluorescence. When fused with lysosomes in late stage, the acidic autolysosomes degrade EGFP and emit red fluorescence.^20^ The tfLC3 plasmids were transfected by using X-tremeGENE HP DNA Transfection Reagent (Roche, Mannheim, Germany) following the company’s manual.

Transfected cells were incubated at 37 °C in a CO_2_ incubator for 24 h. The transfected cells were washed with PBS and infected with *E. coli* O157:H7 EDL933 WT strain as described above. Cells were fixed with 4% paraformaldehyde for 30 min at room temperature and mounted with Fluoro-gel with DAPI (Electron Microscopy Sciences, Hatfield, PA, USA). Red and yellow puncta were visualized by using inverted EVOS FL fluorescence microscope (Life Technologies) by counting a total of >20 cells for each treatment.^20^

### Immunoblotting

Immunoblotting analysis was conducted according to procedures previously described.^53^ Antibodies against LC3B, caspase-3, PARP, IRE1-*α*, p-ERK/ERK, p-Akt/Akt, and p-PKA/PKA were purchased from Cell Signaling Technology (Beverly, MA, USA). Anti-*β*-actin antibody was purchased from Developmental Studies Hybridoma Bank (DSHB, Iowa City, IA, USA). Binding of antibodies was detected using HRP-coupled anti-rabbit or anti-mouse immunoglobulin (Cell Signaling Technology), and visualized using Pierce ECL Western Blotting Substrate (ThermoFisher, Waltham, MA, USA). Density of bands was quantified, and then normalized with *β*-actin as loading control.

### Bacterial supernatant cytotoxicity assay

Vero cells were seeded to a 96-well plate and grown at 37 °C with 5% CO_2_ for 24 h. The cell monolayers were washed with serum-free DMEM once and 200 *μ*l of diluted bacterial supernatants (1 : 1 diluted with DMEM) were added into corresponding wells. Then, cells were incubated at 37 °C for 12 h and 100 *μ*l supernatant of each well was transferred into a new 96-well plate. The lactate dehydrogenase (LDH) activity was determined using the Cytotoxicity Detection Kit (LDH) from Roche (Indianapolis, IN, USA) according to the manufacturer’s instructions. The absorbance was measured at 490 nm using a microplate reader (BioTek Synergy H1, VT).

### PKA inhibition assay

HT-29 cells (1×10^6^ cells per well) were seeded in 12-well plates and incubated for 24 h. Cells were challenged with 10^7^ CFU/ml *E. coli* O157:H7 EDL933 WT strain and coincubated with/without PKA inhibitor (H89, Cell Signaling Technology). HT-29 cells were collected for protein extraction at 4 h following *E. coli* O157:H7 infection at 37 °C with 5% CO_2_.

### cAMP measurement

After *E. coli* O157:H7 EDL933 WT strain infection, cells were washed once with HPLC grade water, 0.8 M perchloric acid (Sigma) was added, and cells were collected. Cell lysates were incubated on ice for 10 min and then sonicated for 3 min. Cell lysates were centrifuged (2000×*g*, 5 min), and the supernatant was neutralized with 2.2 M KOH. The samples were centrifuged again for 10 min at 10 000×*g*, and the resulting supernatants were injected onto the Shimadzu HPLC series (LC-20AD, equipped with a diode-assay detector (SPD-M30A), SHIMADZU, Kyoto, Japan) with Luna C18 column (150×4.6 mm i.d.; 3 *μ*m particle size) (Phenomenex, Torrance, CA, USA). The mobile phase consisted of 60 mM K_2_HPO_4_ and 40 mM KH_2_PO_4_ (elute A) and 100% methanol (elute B). The gradient elution was at a flow rate of 0.5 ml/min using the following program: the initial ratio of B was 0% and kept for 2 min; raised to 10% in 2 min, 20% in 8 min and held for 3 min, and then returned to 10% within 0.5 min and stopped at 16 min. Detection was performed by a SPD-M30A detector set at 260 nm at 25 °C. Amounts were quantified using peak areas and the peak identification was confirmed using cAMP standard (Sigma).

For cAMP ELISA assay, infected cell monolayers were lysed with 200 *μ*l 0.1 M HCl and incubated for 10 min at room temperature. The lysates were centrifuged at 600×*g* for 10 min and the supernatants were assayed according to the manufacturer’s procedure (Direct cAMP ELISA kit, Enzo Life Sciences, Farmingdale, NY, USA). Cells treated with 50 *μ*M forskolin (Enzo Life Sciences) for 15 min were used as a positive control.

### Statistical analysis

Statistical analyses were conducted as previously described.^53^ Data were analyzed as a complete randomized design using GLM (General Linear Model of Statistical Analysis System, SAS, 2000, Cary, NC, USA). All data were analyzed by one-way ANOVA and two-tailed Student’s *t*-test. Means±S.E.M. are reported. A *P-*value ≤0.05 was considered as significant.

## Additional information

**Publisher’s note:** Springer Nature remains neutral with regard to jurisdictional claims in published maps and institutional affiliations.
