# Supplementary material for: Escherichia coli O157:H7 suppresses host autophagy and promotes epithelial adhesion via Tir-mediated and cAMP-independent activation of protein kinase A
Source: Cell Death Discov. 2017 Oct 2;3:17055–. doi: 10.1038/cddiscovery.2017.55 (PMC5624281; doi:10.1038/cddiscovery.2017.55)
Supplement: Supplementary Information [file cddiscovery201755-s1.docx]

**SUPPLEMENTARY INFORMATION**

**Bacterial internalization assay.** Bacterial internalization was conducted as described in previous publication ^1^. Briefly, HT-29 cells (5×10^5^ cells per well) were seeded in a 24-well plate, cultured until 80-90% confluence. Cells were infected with *E. coli* O157:H7 EDL933 WT strain for 4 h. After infection, the cell monolayers were washed 3 times with PBS, treated with 0.5 ml of fresh DMEM media supplemented with 10% FBS and 100 μg/ml of gentamicin and incubated at 5% CO_2_ incubator for an additional 2 h to kill the extracellular attached *E. coli* O157:H7. The monolayers were washed 3 times with PBS and lysed with 0.6 ml 0.1% Triton X-100 in PBS. The 100 μl of bacterial suspension was plated on LB agar in duplicate, and the bacterial colonies were counted after 24 h of incubation at 37°C. The internalization level was expressed as the number of CFU recovered post gentamicin treatment per well or as a percentage of the initial inoculum.

**Bacterial adhesion assay with** **choloroquine treatment.** Bacterial adhesion assay was conducted as described in our previous publication ^2^. Briefly, HT-29 cells (5×10^5^ cells per well) were seeded in a 24-well plate, cultured until 80-90% confluence. For chloroquine treatment, cells were infected with *E. coli* O157:H7 EDL933 WT strain and co-incubated with 10 or 20 μM chloroquine for 4 h. After infection, the cell monolayers were washed 3 times with PBS, and lysed with 0.1% Triton X-100 in PBS. The cell lysate was serial diluted and plated on LB agar in duplicate. The bacterial colonies were enumerated after 24 h of incubation at 37°C. The percentage of adhesion was calculated by dividing the number of colonies (recovered *E. coli* O157:H7) by the initial number of bacteria added and multiplied by 100.

**Quantitative reverse transcription PCR (qRT-PCR) analysis.** Total RNA was extracted from HT-29 cells 4 h post *E. coli* O157:H7 infection using RNeasy Mini Kit (Qiagen, Valencia, CA) and reverse transcribed using a iScript^TM^ kit (Bio-Rad, Hercules, CA). cDNAs were used as a template for qRT-PCR analysis of selected genes using a CFX96™ Real-Time PCR Detection System (Bio-Rad). SYBR Green Master Mix (Bio-Rad) was used for all qRT-PCR reactions. Primers for qRT-PCR are listed in the Table S1. β-actin was used as the housekeeping gene. The amplification efficiency was 0.90-0.99 ^2^.

**Supplementary tables:**

**Table S1. Internalization of *E. coli* O157:H7 by HT-29 cells (n = 6)**

| Strain  *E. coli* O157:H7 | Cell-associated^1^  (CFU×10^6^/well) | Internalized^2^  (CFU/well) | %  Internalization^3^ |
| --- | --- | --- | --- |
| 1 | 6.78 | 305 | 6.55E-05 |
| 2 | 6.56 | 250 | 5.36E-05 |
| 3 | 6.09 | 440 | 9.44E-05 |
| 4 | 6.95 | 290 | 6.22E-05 |
| 5 | 6.64 | 215 | 4.61E-05 |
| 6 | 6.32 | 220 | 4.72E-05 |

^1^Total associated bacteria (including attached and internalized bacteria) after washing; ^2^Internalized bacteria after washing and gentamycin treatment; ^3^Percentage of inoculum internalized into host cells.

**Table S2. Primer sets used for quantitative RT-PCR in human cells**

| **Gene Name** | **Accession No.** | **Product Size** | **Direction** | **Sequence (5’🡪3’)** | **Source** |
| --- | --- | --- | --- | --- | --- |
| *ERK1* | NM_002746.2 | 191bp | Forward | CAAGTCAGACTCCAAAGCCCT | This study |
|  |  |  | Reverse | TCAGCCGCTCCTTAGGTAGG |  |
| *ERK2* | NM_002745.4 | 133bp | Forward | ATTTGTCAGGACAAGGGCTCA | This study |
|  |  |  | Reverse | CCTCCAAACGGCTCAAAGGA |  |
| *IRE1-α* | NM_001433.3 | 210bp | Forward | CGGCCTCGGGATTTTTGGAA | This study |
|  |  |  | Reverse | AGCGTATACAGGCTGCCATC |  |
| *ACTB* | NM_001101.3 | 100bp | Forward | GATGAGATTGGCATGGCTTT | ^3^ |
|  |  |  | Reverse | CACCTTCACCGTTCCAGTTT |  |

**Supplementary Figures:**

**Figure S1**. **Inhibition of autophagy increased *E. coli* O157:H7 adhesion.** HT-29 cells were infected with *E. coli* O157:H7 EDL933 WT strain in presence of 10 or 20 µM of chloroquine during 4 h infection. Means ± SEM; n=6. **, *P* ≤ 0.01; *, *P* ≤ 0.05.

**
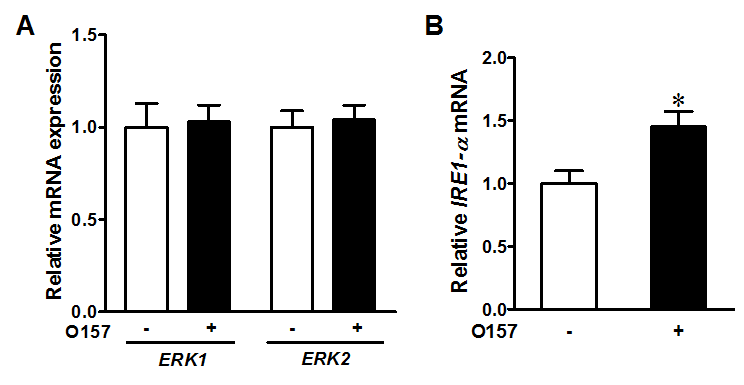
**

**Figure S2**. **mRNA expression of ERK1, ERK2 and IRE1-α in HT-29 cells with or without *E. coli* O157:H7 infection.** (A) mRNA expressions of *ERK1* and *ERK2*; (B) *IRE1-α* mRNA expression. HT-29 cells were collected for total RNA extraction following 4 h infection of *E. coli* O157:H7 EDL933 WT strain. Means ± SEM; n=4. *, *P* ≤0.05.

**Reference:**

1. Sheng HQ, Wang J, Lim JY, Davitt C, Minnich SA, Hovde CJ. Internalization of Escherichia coli O157:H7 by bovine rectal epithelial cells. *Front Microbiol* 2011; **2**.

2. Xue Y, Zhang H, Wang H, Hu J, Du M, Zhu MJ. Host inflammatory response inhibits *Escherichia coli* O157:H7 adhesion to gut epithelium through augmentation of mucin expression. *Infect Immun* 2014; **82:** 1921-1930.

3. Xue Y, Zhang H, Sun X, Zhu MJ. Metformin improves ileal epithelial barrier function in interleukin-10 deficient mice. *Plos One* 2016; **11:** e0168670.
